# Genotype imputation accuracy in a F2 pig population using high density and low density SNP panels

**DOI:** 10.1186/1471-2156-14-38

**Published:** 2013-05-08

**Authors:** Jose L Gualdrón Duarte, Ronald O Bates, Catherine W Ernst, Nancy E Raney, Rodolfo JC Cantet, Juan P Steibel

**Affiliations:** 1 Department of Animal Science, Michigan State University, East Lansing, Michigan, USA; 2 Departamento de Producción Animal, Facultad de Agronomía, UBA-CONICET, Buenos Aires, Argentina; 3 Department of Fisheries and Wildlife, Michigan State University, East Lansing, Michigan, USA

## Abstract

**Background:**

F_2_ resource populations have been used extensively to map QTL segregating between pig breeds. A limitation associated with the use of these resource populations for fine mapping of QTL is the reduced number of founding individuals and recombinations of founding haplotypes occurring in the population. These limitations, however, become advantageous when attempting to impute unobserved genotypes using within family segregation information. A trade-off would be to re-type F_2_ populations using high density SNP panels for founding individuals and low density panels (tagSNP) in F_2_ individuals followed by imputation. Subsequently a combined meta-analysis of several populations would provide adequate power and resolution for QTL mapping, and could be achieved at relatively low cost. Such a strategy allows the wealth of phenotypic information that has previously been obtained on experimental resource populations to be further mined for QTL identification. In this study we used experimental and simulated high density genotypes (HD-60K) from an F_2_ cross to estimate imputation accuracy under several genotyping scenarios.

**Results:**

Selection of tagSNP using physical distance or linkage disequilibrium information produced similar imputation accuracies. In particular, tagSNP sets averaging 1 SNP every 2.1 Mb (1,200 SNP genome-wide) yielded imputation accuracies (*IA*) close to 0.97. If instead of using custom panels, the commercially available 9K chip is used in the F_2_, *IA* reaches 0.99. In order to attain such high imputation accuracy the F_0_ and F_1_ generations should be genotyped at high density. Alternatively, when only the F_0_ is genotyped at HD, while F_1_ and F_2_ are genotyped with a 9K panel, *IA* drops to 0.90.

**Conclusions:**

Combining 60K and 9K panels with imputation in F_2_ populations is an appealing strategy to re-genotype existing populations at a fraction of the cost.

## Background

The search for regions in the genome containing genetic variants that affect production traits requires experimental populations to identify the segregating QTL within and between parental populations [1]. The F_2_ design is commonly used to map QTL segregating in divergent parental lines [2,3]. To produce reliable analyses of association or genetic evaluations using genomic information, a great number of individuals with phenotypes and high density (HD) genotypes are required [4]. However, HD genotypes for large numbers of animals are expensive to obtain [5,6]. A way of reducing cost is to genotype individuals from base generations (parents) in HD, and their more numerous descendants at low density (LowD) [6,7]. Then, using selected SNP from the HD panel, called tagSNP, the non-typed SNP are imputed with high accuracy [7]. Imputing HD genotypes of progeny from LowD genotypes, conditional on grandparental and parental HD genotypes, may result in higher imputation accuracies than those obtained using a reference panel from unrelated individuals [7-9]. This is because HD genotypes from base generations can be traced within family by means of co-segregation or descendant probabilities [6] while searching for the phase of parental alleles [7].

Most studies on genotype imputation of livestock species have been performed with purebreds [4,7,9-13], and genotype imputation from crossbreds has been largely absent. With regard to agricultural plant species, studies on genotype imputation have used inbred lines [8], recombinant inbred lines (RILs) in Nested Association Mapping (NAM) designs [14,15], and Multiparent Advanced Generation Inter-Cross studies (MAGIC) [16]. Genotype imputation has also been employed in human studies of genome-linkage analysis for test association of candidate transcriptional regulators with gene expression [17]; and also in a model organism in biomedical research such as the mouse, imputation of genotypes from crosses of inbred lines was used to identify candidates genes for complex disease [18,19].

Imputing genotypes in humans, plants, livestock, or model organisms, is similar in the sense that a small number of founding individuals can be genotyped at high density, and the bulk of the mapping population can be genotyped at low density using linkage information. In this paper we focus on imputing F_2_ individuals from a three generation (F_0_, F_1_ and F_2_) population of Duroc × Pietrain crossbred pigs. The F_0_ and F_1_ animals were genotyped in HD (60K). The F_0_ populations used to map QTL in pigs are typically composed of a small number of animals (in our case, 4 males and 15 females) [1,20-22]. As it is expected that few recombinations occur in the first generations, these populations have low resolution to map QTL [23]. However, and for the same reason, there is a potential for attaining high accuracy of imputation. The latter effect can be taken to advantage for imputing HD genotypes from inexpensive LowD F_2_ genotypes, which subsequently allows combining existing data from experimental populations in a meta-analysis for association. There are several reasons for this strategy to be attractive. First, several of these populations have been recently created [21,22,24,25] and DNA from these animals is available. Second, extensive datasets of phenotypes have been recorded for these populations including for traits that are expensive or difficult to measure, such as the content of intramuscular fat and composition of fatty acids [25], age at puberty in gilts [22], and meat tenderness [26]. Finally, these populations are generally developed from breeds that are divergent for some traits of interest such as fat/lean content, meat quality or reproductive efficiency, take for example: Duroc × Pietrain [1,21], Duroc × Landrace [24], Duroc × Large-White [25], White-Duroc × Erhualian [22], Meishan × Duroc [27], Berkshire × Duroc [20].

Therefore, it follows that imputation of F_2_ LowD to HD genotypes with high accuracy would be useful and convenient, providing a cost effective strategy as a first step for association analyses or meta-analyses. Different methods have been employed to select tagSNP in LowD panels. Two of them are: 1) imposing restrictions on the minimum value of linkage disequilibrium (LD) or *r*_*t*_^2^ between markers [28], 2) selection of tagSNP that are evenly spaced using the physical distance between markers[4,11,12]. In addition, commercial chips are also available with medium density segregating SNP selected from several populations, as for example for bovine [29] and pig [10]. A question arises of how many SNP are needed to attain a high accuracy of imputation for a given F_2_ population. Another question is whether a specific chip has to be custom designed, or whether current commercially available chips can be used. Finally, it is important to determine whether both the F_0_ and F_1_ have to be genotyped at HD, or if just genotyping the F_0_ is adequate to obtain a high accuracy of imputation in the F_2_.

The goal of this research was to estimate the accuracy of imputation at HD (60K), from LowD F_2_ genotypes for a Duroc × Pietrain population, using different genotyping schemes. The strategies were evaluated by means of Monte Carlo simulation, conditional on the genotypes from animals in the first two generations (F_0_ and F_1_). In doing so, two methods of tagSNP selection were considered and their results were compared to those obtained from a commercial panel chip (9K). In addition to simulations, accuracy of imputation was evaluated using experimental data, taking advantage of a reduced number of F_2_ animals that were genotyped at HD.

## Results

### Linkage disequilibrium and selection of tagSNP

Table 1 displays the number of tagSNP selected with different values of LD in an intermediate size chromosome (SSC12), reflected by the measure *r*_*t*_^2^. As the value of *r*_*t*_^2^ increases, more tagSNP are selected and *IA* increases. As an example, when *r*_*t*_^2^ = 0.2, 79 tagSNP were selected at an average distance of 0.79 Mb and at an accuracy of 0.970. On the other hand for *r*_*t*_^2^ = 0.5, 399 tagSNP were selected, positioned at an average distance of 0.16 Mb with *IA* being equal to 0.982 (Table 1).

**Table 1:** **Accuracy of imputation using tagSNP selected for different values of r**_**t**_^**2 **^**on chromosome 12**

**r**_**t**_^**2**^	**Number of tagSNP**	**Number of SNP genomewide**	**Average distance between SNP (Mb)**	**Imputation accuracy ( *IA *)**
0.1	33	1295	1.86	0.960
0.2	79	3100	0.79	0.970
0.3	158	6199	0.40	0.976
0.4	266	10436	0.24	0.980
0.5	399	15654	0.16	0.982

*r*_*t*_^2^ = Threshold of LD in statistical selection, Number of tagSNP = Number of SNP selected for a particular *r*_*t*_^2^, Number of SNP Genomewide = The equivalent number of SNP that are needed genome-wide to keep the same average inter-marker distance. Average distance between SNP (Mb) = Average distance between tagSNP selected, Imputation accuracy (*IA*) = Imputation accuracy of non-tagSNP. Results using simulated data.

### Evenly spaced SNP

**Figure 1:**
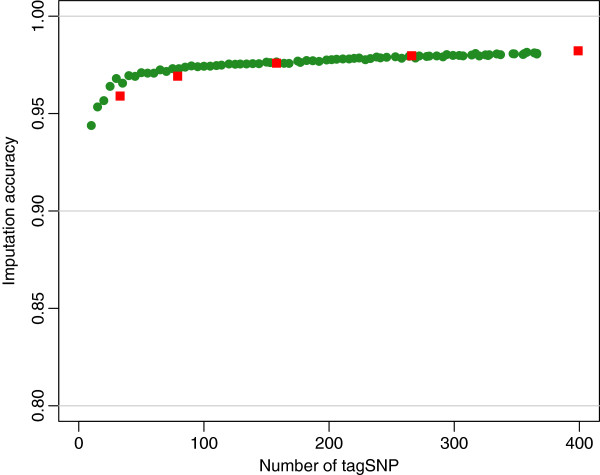
**Accuracy of imputation (IA) using tagSNP selected using LD information or evenly spaced.** Imputation accuracy as a function of number of tagSNP selected: evenly spaced tagSNP panels (green dots), tagSNP-LD panels (red squares). Results shown correspond to simulated data from chromosome 12.

**Figure 2:**
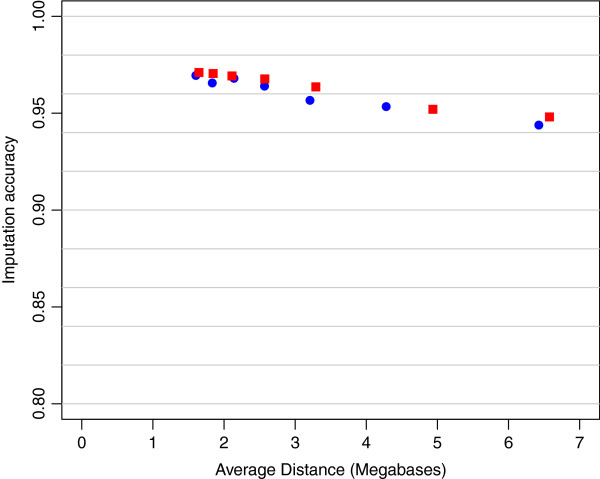
**Accuracy of imputation (IA) as a function of average tagSNP distance for evenly spaced panels on chromosomes 1 and 12.** Imputation accuracy as a function of the average distance between evenly spaced tagSNP in megabases (Mb) for seven SNP panels on simulated data from chromosome 1 (red squares) and chromosome 12 (blue dots).

The *IA* using tagSNP selected using either LD information or evenly spaced SNP were similar. For example, the *IA* of non-typed SNP on SSC12 were 0.973 and 0.970, respectively, for 80 evenly spaced SNP as compared with 79 tagSNP selected with *r*_*t*_^2^ = 0.2 (Figure 1). Results for other densities of tagSNP were similar (Figure 1). Moreover, evenly spaced tagSNP sets of comparable density across chromosomes yielded similar accuracies. Thus, for example an average inter–marker distance of 2.1 Mb, 140 tagSNP on chromosome 1 and 30 tagSNP on chromosome 12 produced *IA* of 0.969 and 0.968, respectively (Figure 2). In summary, a minimum of 1,200 evenly spaced tagSNP across the genome (average distance = 2.1 Mb) are needed in this F_2_ population to attain imputation accuracy *IA* ≥ 0.97 when the F_0_ and F_1_ are genotyped with a SNP60 chip.

### Imputed genotypes in experimental F2 animals

### 9K commercial chip

The values of *IA* were calculated for two scenarios and for each chromosome, using a 9K SNP list that was developed for producing a commercial LowD panel (GeneSeek, Inc., Lincoln, NE, USA; described in Badke et al. [10]).

**Figure 3:**
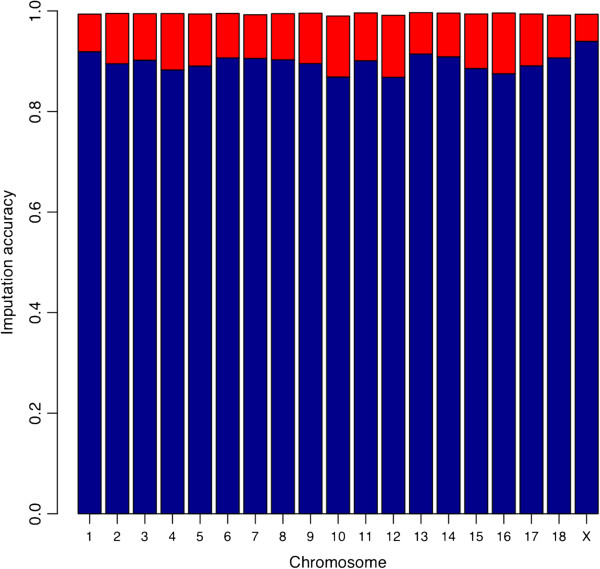
**Accuracy of imputation (IA) for SNP on 60K chip using the 9K panel as tagSNP.** Average accuracy of imputation for each chromosome using experimental data: Blue bars correspond to the case of F_0_ at high density (HD), F_1_ and F_2_ at low density (LowD). Red bars correspond to gain in accuracy of imputation when the F_1_ is genotyped at HD.

**Figure 4:**
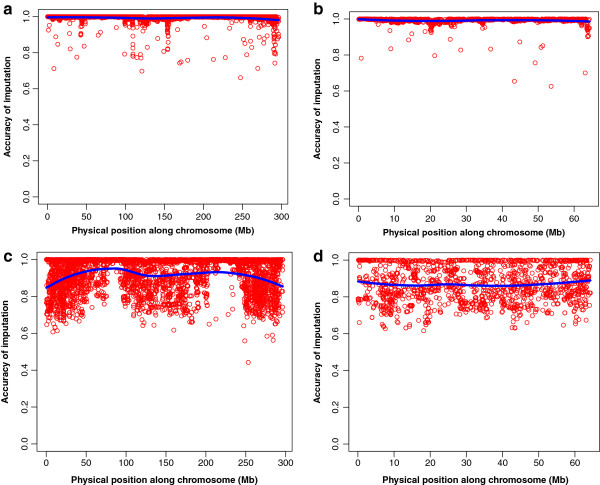
**Accuracy of imputation (IA) across chromosomes 1 and 12 under two genotyping scenarios.** Generation F_0_ and F_1_ at high density, F_2_ at low density: chromosome 1 (**a**), chromosome 12 (**b**). Generation F_0_ at high density, F_1_ and F_2_ at low density: chromosome 1 (**c**), chromosome 12 (**d**). The blue line displays a local regression fit of the data. All results were obtained using the experimental data.

**Figure 5:**
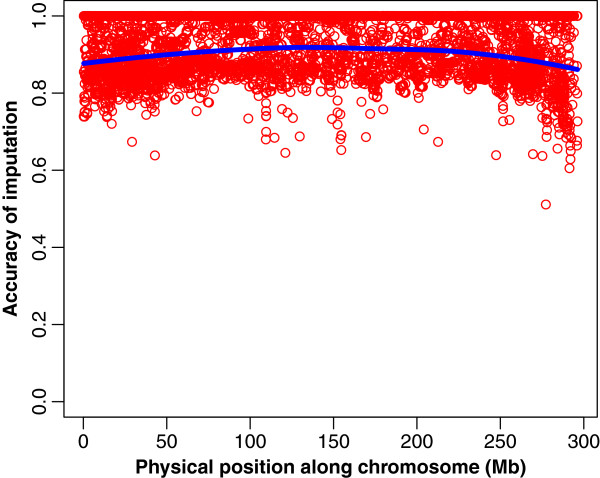
**Accuracy of imputation (IA) per SNP ignoring pedigree relationship on chromosome 1.** Imputation accuracy of experimental data as a function of the chromosomal positions of SNP using information on LD only. Generation F_0_ and F_1_ genotyped at high density and F_2_ genotyped at low density with the relationships between F_0,_ F_1_ and F_2_ omitted.

Imputation accuracies *IA* were 0.90 and 0.99 when the F_1_ was genotyped at low or high density, respectively (Figure 3). In the latter case, although the accuracy was high in all chromosomes (0.99), SNP in some regions were imputed with lower accuracy (Figure 4). High *IA* in the F_2_ were obtained across all SNP when the F_1_ was genotyped at HD (Figure 4a,b). However, when the F_1_ was genotyped at LowD, *IA* in F_2_ individuals decreased along the whole chromosome (Figure 4c,d). A logical question to consider is the following: how much accuracy is gained when including pedigree information, when compared with the use of population-wise LD as the unique source of information? To answer this, the imputation was performed again using as reference panel the genotypes of F_0_ and F_1_ animals and the F_2_ at LowD, but without specifying the pedigree of the F_2_s. In other words, the F_2_ animals were assumed unrelated and their parents were unknown. For chromosome 1 the results are displayed in Figure 5. Notice that the average *IA* in the F_2_ was equal to 0.90. Therefore, the *IA* was lower than when the information on relationships was used (0.99, Figure 4a,b). This indicates that the inclusion of HD genotypes from related animals and explicitly specifying paternities greatly increases accuracy of imputation.

The *IA* from both genotyping scenarios (Figures 3 and 4) reflect an average drop of 0.1 when the F_1_ is genotyped at LowD. To gain further insight, the simulated haplotypes of two families were used to calculate accuracy of imputation in each scenario. When the F_1_ is genotyped at LowD, the results showed that the phase error among the SNP that are not tagSNP increased. This loss of accuracy in determining the SNP phase can be traced back to the F_0_ generation in which the non-tagSNP are also phased with low accuracy. Furthermore, the proportion of SNP with uncertain phase in the F_1_ genotyped at HD was 4%, and the ensuing accuracy of haplotyping was 0.97. However, when the F_1_ was genotyped at LowD the proportion of SNP with uncertain phase increased to 30%, and the corresponding accuracy of haplotyping for the non-tagSNP of F_1_ genotypes dropped to 0.85. In a further analysis with the F_1_ generation genotyped at HD and used as a reference population (ignoring F_0_ genotypes), this resulted in 43% of non-tagSNP with uncertain phase in the F_1_ at HD, and the haplotyping accuracy was even lower (0.78). These results suggest that, in order to have a high accuracy of imputation for non-tagSNP in F_2_ genotypes, certainty of the phase in the F_1_ genotypes is required. Such accurately estimated phase is guaranteed when two generations of HD genotypes (F_0_, F_1_) are available.

A closer look at Figures 4 and 5 indicates that the position of the SNP had some effect over *IA*. Therefore, we investigated the relationship between single SNP imputation accuracy and each SNP’s MAF, distance to the nearest tagSNP, and allelic frequency difference between founding breeds.

### Minor allele frequency (MAF)

The measure of accuracy based on counting the number of alleles correctly imputed is sensitive to the allelic frequency [8,12,30]. In the current study, the square of the correlation (*R*^*2*^) between observed and imputed genotypes was used as a robust measure of accuracy of imputation. It is worth noting that the scale of this measure is somewhat different from the one derived from *AI* (Table 2).

**Table 2:** **Imputation accuracy of SNP on chromosome 12 measured by IA or by R**^**2**^

**Scenario**	**Genotype design**			**Accuracy of imputation**	
	**Grandparents**	**Parents**	**Progeny**	***IA***	***R***^***2***^
1	HD	HD	LowD	0.962	0.884
2	HD	LowD	LowD	0.833	0.408

Comparison of two measures of accuracy of imputation calculated using experimental data (tagSNP panel = 30 SNP), under two situations: 1) F_0_ and F_1_ at high density (HD), F_2_ at low density (LowD), 2) F_0_ at HD, F_1_ and F_2_ at LowD.

### MAF using the 9K panel in the F_2_

**Figure 6:**
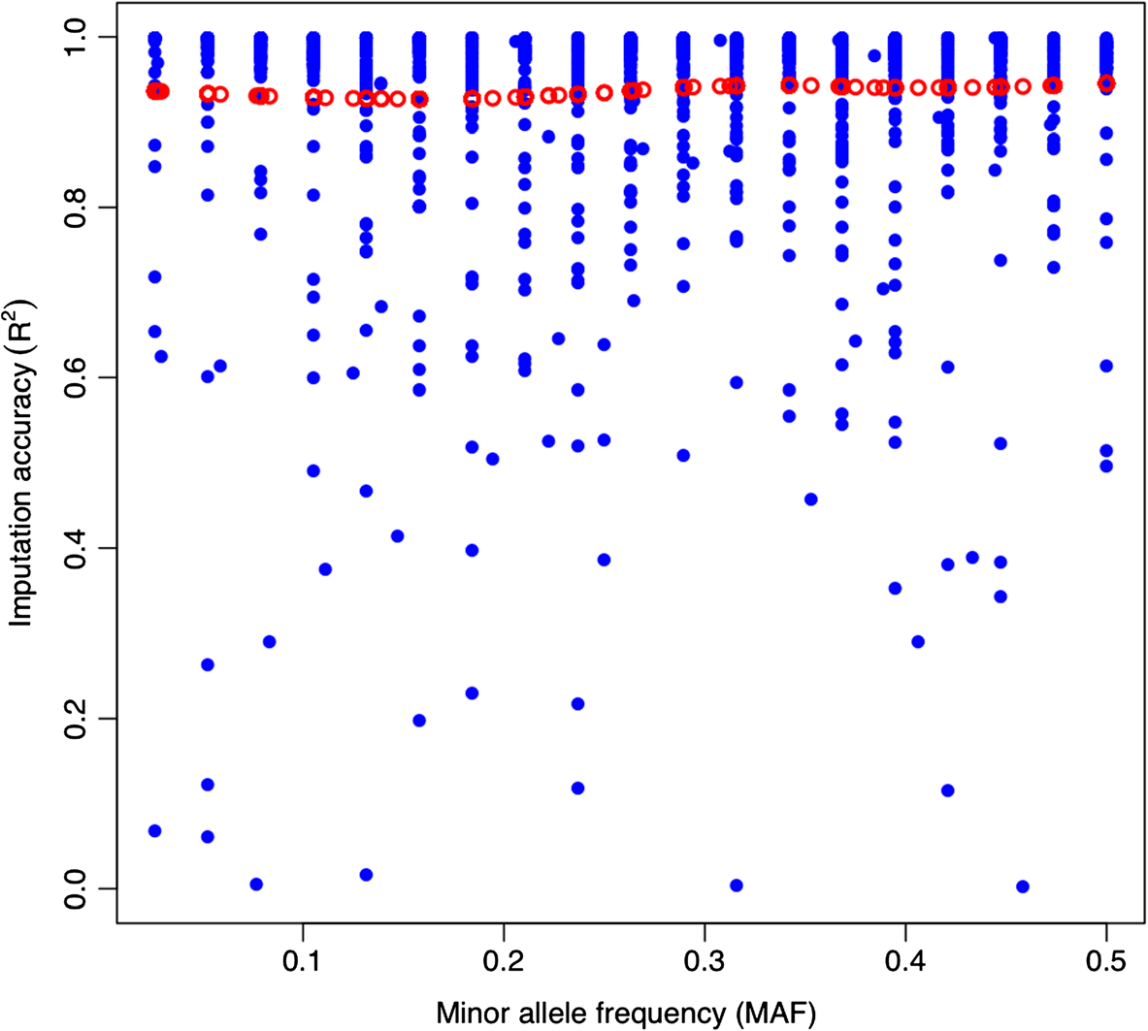
**Imputation accuracy (R**^**2**^**) for SNP on chromosome 12 as a function of the minor allele frequency in the F**_**0**_**.** Accuracy of imputation of experimental data as a function of minor allele frequency of each SNP (blue dots). Local regression fit (red dots).

Figure 6 shows that the MAF of the imputed SNP was not related to *R*^*2*^ in these data. Notice also that alleles with extreme frequencies (MAF < 0.1) can be imputed with accuracy similar to those SNP at intermediate frequencies (MAF > 0.3).

### Distance to the closest tagSNP

**Figure 7:**
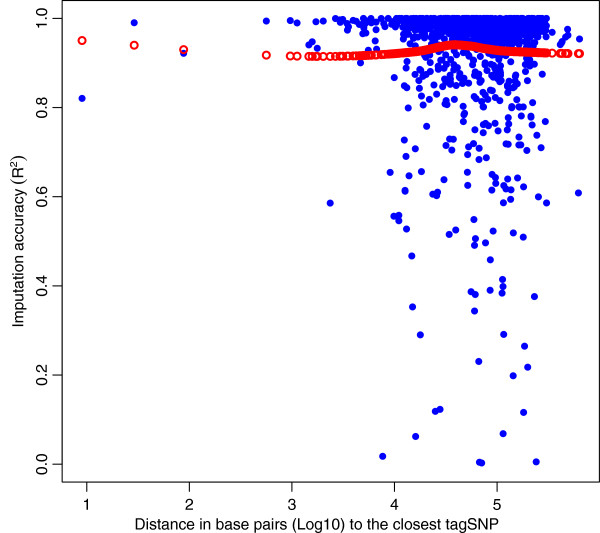
**Imputation accuracy (R**^**2**^**) for SNP on chromosome 12 as a function of the distance to the closest tagSNP.** Blue dots are non-tagSNP (Experimental data); distance in base pairs (Log10). Local regression fit is displayed by the red dots.

No differences in *R*^*2*^ were found for the range of distances between non-tagSNP and tagSNP observed (average was equal to 0.936 Mb). Therefore, for an average density between tagSNP of 0.26 Mb, *R*^*2*^ is similar for a SNP that is in the middle of the interval than for a SNP that is close to the tagSNP (Figure 7). This observation suggests that the density of tagSNP was enough to attain a reasonably equal *R*^*2*^ for all SNP within the interval.

### Effect of the difference in allelic frequencies in the F_0_

**Figure 8:**
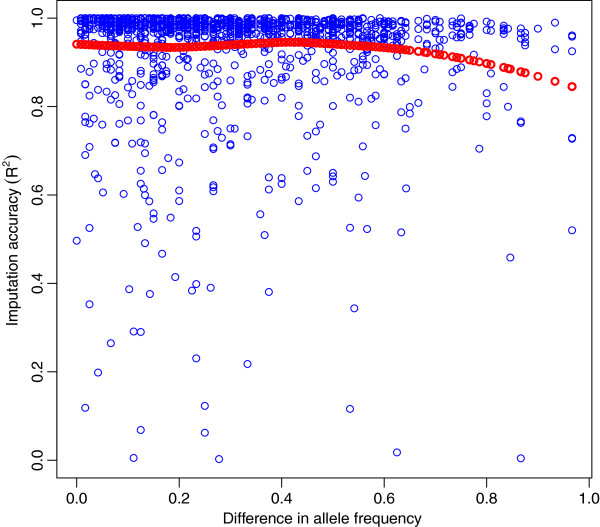
**Imputation accuracy (R**^**2**^**) for SNP on chromosome 12 as a function of the difference in allelic frequencies.** Accuracy of imputation using experimental data for the difference in allelic frequencies (blue circles) between founding breeds (Pietrain and Duroc). Local regression fit (red dots).

The difference in allelic frequency between founding populations does not seem to affect the *R*^*2*^. This means that even SNP that segregate at very different frequencies in founders can be imputed with high accuracy as revealed in Figure 8. Moreover, the apparent drop in *R*^*2*^ for MAF differences over 0.75 presented in Figure 8 is largely an artefact of very small number of SNP used in the smoothing line fit.

## Discussion

### SNP selection methods and accuracy of imputation

A main goal of the present research was to evaluate accuracy of imputation in an F_2_ cross of pigs (Duroc × Pietrain) using different genotyping scenarios. In a first stage, *IA* was calculated from simulated F_2_ data. An ideal situation for linkage based imputation would be to select SNP equally spaced based on genetic distance, as the possibility of recombination between imputed SNP and tagSNP would be minimal. However, this is not possible in the absence of a high resolution linkage map. Consequently, to position the tagSNP we used two proxies: a) physical spacing, and b) LD-based selection. For our simulated population, the two proxies produced the same results, most likely because it was assumed that 1cM = 1 Mb uniform recombination rate. Therefore, in this simulated population, the average distance between tagSNP throughout the genome proved to be a good indicator of accuracy of imputation (*IA*), as values greater or equal to 0.97 were obtained using average distances among tagSNP that were less than or equal to 2.1 Mb. Next, the selection of tagSNP using the LD method was compared to choosing SNP located at regularly spaced intervals throughout the genome. In the first method, LD was measured by *r*_*t*_^2^, the minimum threshold of *r*^2^ between any non-tagSNP with at least one tagSNP. It was observed that when *r*_*t*_^2^ increased, the number of selected tagSNP and *IA* also increased. The accuracy was between 0.960 (*r*_*t*_^2^ = 0.1) and 0.982 (*r*_*t*_^2^ = 0.5), with average distance between tagSNP of 1.86 Mb and 0.16 Mb, respectively. Xu et al. [28] used *r*_*t*_^2^ = 0.8 to select a set of tagSNP for genome-wide association analyses in humans. Their use was slightly different from ours in that they were selecting SNP to tag causative variants for genome-wide association using population level LD information only. On the other side, we wanted to use this method to select SNP that were more evenly spaced in terms of genetics distance as done previously with outbred pig populations [10], but this time exploiting within and between family LD. Consequently, low levels of *r*_*t*_^2^ were used in the current study as we found that with a threshold of *r*_*t*_^2^ ≥ 0.6, many tagSNP were selected with marginal increases of *IA*. The second method employed to select tagSNP consisted of dividing the chromosome into segments of equal size, and then choosing the SNP that lay closest to the center of the segment. Other studies have used evenly spaced tagSNP by selecting one SNP every given number of markers [12], or by choosing in each segment the SNP with the largest MAF [4],11,12]. The fact that we had available a sizable number of SNP throughout the genome, i.e. 60 K, made it possible to select approximately evenly spaced SNP with a wide range of MAF, as long as those SNP were segregating in the population. The values of *IA* calculated while using tagSNP chosen at evenly spaced segments were similar to those obtained using the LD method. This similarity of results may be due to an assumption made in the method of SNP selection at evenly spaced intervals, i.e. that the distribution of LD along the genome is almost uniform and there are no large blocks of LD. In the current research, the haplotypes of F_1_ animals are sampled from two populations: Duroc and Pietrain. The resulting LD was relatively high and uniformly distributed, except for a few blocks with extremely high LD: blocks with at least 7 consecutive SNP with *r*^2^ ≥ 0.8. For this reason, evenly spaced tagSNP and tagSNP selected based on the LD method produced similar imputation accuracy at equivalent density. Although we indeed simulated assuming uniform recombination rates, these results seem to agree also with experimental data, where the two methods of selection used here produced virtually the same accuracy in an outbred pig population [10]. Designing custom low density SNP panels for each population of interest would not be cost effective. Consequently, we investigated the imputation accuracy obtained using a commercially available SNP chip with markers selected based on physical position and MAF [10].

### Imputation using 9K panel and genotyping scenarios

Data from a 9K chip (average distance between SNP = 0.30 Mb) were used as a LowD panel to impute to a HD 60K panel. Using the experimental data from F_2_ individuals, different genotyping scenarios were tested. In the first scenario, data consisted of F_0_ and F_1_ genotypes at HD and F_2_ at LowD, and average *IA* was 0.99. Similarly, Weigel et al. [13] imputed 8K genotypes to 43K using information of the sire, dam, and grandsires (paternal and maternal), and obtained a value of *IA* > 0.95.

Our second scenario included the F_1_ genotyped at 9K, between the generations of grandparents and grandoffspring, and it was observed that *IA* of F_2_ decreased to 0.9. In our last scenario F_0_ and F_1_ were genotyped at HD and F_2_ at LowD but the relationships between the F_2_ and the reference panel were ignored, resulting in an average accuracy of imputation of 0.9. Badke et al. [10] used the genotypes of a reference population formed by trios to impute genotypes of an unrelated population, and obtained values of *IA* of 0.90 and 0.95 using reference groups of 16 and 64 animals, respectively.

Habier et al. [6] indicated that the reasons for the decay in accuracy of imputation are two-fold: 1) the accuracy of haplotyping the tagSNP flanking the non-tagSNP; 2) the accuracy of haplotyping the imputed non-tagSNP, conditional on a correct haplotyping of the tag-SNP. Therefore, the impact of both factors under the first two scenarios and taking into account the relationships between the individuals in the F_2_ and in the reference population, were evaluated by means of simulated data. Accuracies of haplotyping were calculated as the number of erroneous inference of phase between consecutive heterozygous markers, as in Druet and Georges [31]. In all scenarios, it was observed that the phases of tagSNP were correct, thus the uncertainty was due to the grandparental origin of the non-tagSNP that were flanked by the tagSNP. The next step was to quantify the fraction of non-tagSNP with uncertain phase. When F_0_ and F_1_ were genotyped at HD and F_2_ was genotyped at LowD, the fraction of non-tagSNP with uncertain phase was 4%, whereas this statistic was 30% when the F_0_ was genotyped at HD, and the F_1_ and F_2_ were genotyped at LowD. The corresponding *IA* were 0.97 and 0.85, respectively. These results suggest that accuracies of imputation in the current study were affected by knowledge of the phase of non-tagSNP. Moreover, when the amount of genotypes from related individuals (i.e., F_0_ at HD) increases, the accuracy of haplotyping goes up, and the accuracy of imputation also increases. These results apply to genotyping designs with a pedigree with a small number of founder individuals genotyped in HD and a large number of progeny genotyped in LowD. If the phase is known in the founders, it is easy to accurately follow transmission of chromosomal segments to the remainder of the population using linkage information. In practice, however the phase needs to be ascertained using LD information. Such information is very limited in cases such as our F_0_ because of reduced sample size. In that case, the researchers can follow two paths. First, as presented with large pedigrees, having extra animals from the same founding population(s) can help in using LD to accurately phase those animals. Second, as presented here, two consecutive generations can be genotyped in HD to use the information in grand-parents (F_0_) to accurately phase the parents (F_1_) and then use linkage information to impute genotypes within the progeny (F_2_). For such approaches to work, full pedigree information (three generations) and two generations of HD genotypes are needed. The approach is still cost effective in typical F_2_ populations [6,32]. These results are partially reaffirmed in large pedigree based imputation.

### MAF effect

The measures of accuracy of imputation that are based only on allelic counts are not useful for comparing SNP having different values of MAF. This is due to the fact that imputation errors are highly sensitive to the value of the allelic frequencies [8,12,30]. To overcome this restriction, two alternative measures of accuracy of imputation have been proposed: 1) the correlation between imputed and observed genotypes [8]; and 2) an accuracy of imputation corrected to its expected value [12,30]. The second method consists of adjusting the calculated accuracy of imputation by the difference between the observed accuracy and an estimate of the expected value under random sampling. There are several possible ways of calculating the accuracy under this method. Regardless of the measure being used to calculate the accuracy, a trend for the accuracy of imputation to drop when MAF < 0.15 has been observed. For example, in maize Hickey et al. [8] observed a decrease in *R*^*2*^ when MAF < 0.10, and the drop was higher when the masked genotypes were >84% of total SNP. Similarly, Lin et al. [30] used human data with the correction for expected accuracy and observed a marked decrease in accuracy of imputation when MAF < 0.15. Hayes et al. [12] used the same correction as Lin et al. [30] with sheep data and found highly variable accuracies of imputation but tending to decrease whenever MAF < 0.10. The correlation between observed and imputed genotypes (*R*^*2*^) was employed in the current research to evaluate the effect of MAF on imputation accuracy. Our results showed that markers with MAF < 0.10 in the founders were imputed with reasonably good accuracy in the F_2_ (Figure 6), a result different from those previously discussed. This is not unexpected considering we used both LD and linkage (pedigree information), as sources of information from our crossbred population. Therefore, the allele frequency in the F_0_ does not matter as long as in that generation the two alleles are segregating. Moreover, whenever the F_1_ is genotyped at HD, SNP with low MAF can be observed in the F_0_ and F_1_. Coupled to the fact that all family relationships are known, this simplifies the imputation of F_2_ animals.

### Possible effects in association

In the current research we compared allelic dosage of observed and imputed genotypes to find accurate genotyping design and imputation methods for LowD genotypes in an F_2_ population. Zhen et al. [33] reported that the regression of phenotype on allelic dosage was an accurate method to evaluate QTL effects. Moreover, they observed that when accuracies of imputation were high, the power for the association test was high. For example, accuracies of imputation > 0.95 were associated with values of power > 0.85. In the current study, the accuracy of imputation obtained with the 9K panel was *R*^*2*^ *=* 0.94, which suggests that the power for an association test is high. Other studies also found that imputation improved the power for association tests. Using data from humans, Hao et al. [34] compared the power for GWAS analysis of four different strategies involving imputation: (1) directly testing for associations using the Illumina 317K SNPs, (2) testing for associations using the entire imputed HapMap SNP set based on the Illumina 317K genotype data; (3) directly testing for associations using the Illumina 650Y SNPs; and (4) testing for associations using the entire imputed HapMap SNP set based on Illumina 650Y genotype data. It was observed that genomic wide imputation (strategies 2 and 4) improved power by 5.5% for the Illumina 317K, or 3.3% for Illumina 650Y, compared to the analyses with assayed SNPs only (strategies 1 and 3, respectively). Similar results were obtained by Anderson et al. [5] for the 300K and 550K platforms.

The cost of genotyping is an important consideration. At present, the cost of commercial HD genotyping (60K) for pigs is more than twice as much as the cost of genotyping with the 9K chip. Assuming a population with a structure similar to the one used here (approximately 20 F_0_, 56 F_1_ and 1000 F_2_), one can genotype 1.9 times more individuals in a scenario with F_0_ and F_1_ at HD, and F_2_ at LowD than in a scenario with F_0_, F_1_ and F_2_ at HD. The imputed genotypes can then be used for association or for meta-analysis studies.

## Conclusions

Designing custom SNP panels for each F_2_ population to be imputed will likely not be cost effective due to the relatively large number of SNP needed to attain reasonable imputation accuracies, and the high development costs for each SNP panel. In particular, for our population we would need a minimum of M = 1,200 markers with average distance of 2.1 Mb to have *IA* over 0.97 in the F_2_. On the other hand, using the 9K panel as tagSNP (LowD) resulted in *IA* of 0.99 when the F_0_ and F_1_ were genotyped at HD and the F_2_ at LowD. The cost of such genotyping scheme would be less than half the cost of using HD genotypes for all individuals. The correlation between observed and imputed genotypes was high (*R*^*2*^ = 0.94), so that the power for future association studies would be high. Thus, under a genotyping strategy of high accuracy of imputation (i.e., F_0_ and F_1_ at HD, F_2_ at LowD), information on imputed genotypes from more animals that is similar to that from a HD panel can be obtained at a lower cost. These results apply to the imputation of markers in the SNP60 beadchip, in populations where a small number of founders can be genotyped at HD and phase of parents of imputed animals can be derived with certainty. Translation of LD-based results, on the other hand, are constrained to pig populations showing similar levels of LD as in the founding animals [35].

## Methods

### Animals

The experimental population was raised at the Michigan State University Swine Teaching and Research Farm, East Lansing, MI [1]. Parents from the initial generation (F_0_) were four unrelated Duroc boars mated to 15 Pietrain sows by artificial insemination. From all resulting F_1_ animals, 50 females and 6 males (progeny of 3 F_0_ sires) were selected as parents for the F_2_ generation, by avoiding full or half sib matings. A total of 1,259 F_2_ piglets were born alive from 142 litters out of 11 farrowing groups. Animal protocols were approved by the Michigan State University All University Committee on Animal Use and Care (AUF# 09/03-114-00).

### Genotyping and data editing

DNA was isolated from white blood cells using standard procedures as we have previously described for this population [1]. Quantity and quality of DNA samples were determined using a Qubit fluorometer (Invitrogen by Life Technologies, Carlsbad, CA, USA). The number of genotyped animals was *N =* 411 (4 F_0_ Duroc boars, 15 F_0_ Pietrain sows, 6 F_1_ males, 50 F_1_ females and 336 F_2_ pigs). Genotyping was performed at a commercial laboratory (GeneSeek, a Neogen Company, Lincoln, NE, USA) using the Illumina PorcineSNP60 beadchip [36]. Out of *M* = 62,163 SNP, 6,422 SNP were eliminated as their physical positions were unknown. Mendelian inconsistencies (≤ 0.01%) were taken as missing genotypes, and 12 animals (1 F_1_ and 11 F_2_) with more than 10% of SNP missing were not used in any analysis. By similar consideration, 3,038 SNP were removed from the analyses due to presenting more than 10% missing data. Additionally, 10,139 SNP were excluded as their minor allele frequency (MAF) was below 0.01. These editing policies resulted in a data set comprising 399 pigs with 45,003 SNP per animal. This editing procedure followed that of Badke et al. [35] and the program PLINKv1.07 [37] was used. Additionally, starting with genotypes for F_0_ and F_1_ animals, genotypes for 932 F_2_ animals were simulated conditional on the real pedigree using a gene-dropping model. Simulated genotypes were used to assess alternative tagSNP selection procedures while experimental genotypes on a subset of animals (n = 336) were used to assess imputation accuracy using a SNP list for a 9K commercial chip that has recently been publicly released by GeneSeek Inc. (Lincoln, NE, USA; described in Badke et al. [10]).

### Genotype simulation

A stochastic simulation was performed to evaluate two different methods of selecting tagSNP for imputation on the accuracy of the resulting F_2_ genotypes. The genotypes of 932 F_2_ animals were simulated using *gene-dropping*[38] theory, by conditioning on a real pedigree and on the haplotypes of the 55 F_1_ parents (6 males and 49 females) from the real F_2_ population. The haplotypes were estimated at a high accuracy from the genotypes of the F_1_ parents and 19 F_0_ ancestors (4 Duroc boars and 15 Pietrain sows), using the software MERLIN [39]. The number of recombinations in the F_1_ haplotypes were drawn from a Poisson distribution with mean equal to the length of the given chromosome in Morgans (M) by assuming 1 Mb = 1 cM [40]. The positions of the recombinations were simulated from a uniform distribution using Haldane’s mapping function [41,42]. As an example, there were 1,405 SNP on chromosome 12 that were spread over 64.2 Mb, and the ensuing average distance between markers was 0.04573 Mb. By assuming a recombination rate of 1 cM per Mb [38], the number of recombinations in chromosome 12 was drawn from a Poisson distribution with parameter equal to 64.2 / 100 = 0.642. The next step was to assign the resulting gametes carrying these recombinations of the F_1_ genotypes to their F_2_ progeny.

### TagSNP selection using simulated dataset

Two different methods were used for tagSNP selection: 1) The first one consisted of a statistical search built into the software FESTA [43] and used information on LD [44]. In this method, each SNP was either an element of the tagSNPset, or in LD with an existing element in the tagSNPset, at a value equal or larger than a specified threshold (*r*_*t*_^2^) [10]. A minimum level of *r*_*t*_^*2*^ based on pair-wise LD of the F_1_ haplotypes was selected, so that all SNPs above the chosen threshold were selected as tagSNP. 2) The second method consisted of selecting evenly-spaced markers. The chromosome was divided into *k* segments of equal length, and then the SNP that was closest to the center of the segment was selected. In cases where there were no SNP lying in a segment, no selection was performed resulting in the number of tagSNP≤*k* in segments of approximately equal length.

### Genotype imputation

For simulated data, F_2_ genotypes of non-typed markers were imputed using the algorithm of Lander and Green [39] that predicts the non-tagSNP by conditioning on the observed markers. For computational reasons the pedigree was analyzed on a per litter basis. Thus, for each F_2_ litter, a three generation pedigree was built [45] using the four F_0_ grandparents, the two F_1_ parents, and up to a maximum of 10 F_2_ animals. When the litter had more than 10 progeny, a new “family” was formed with the four F_0_ grandparents, the two F_1_ parents and the remaining F_2_ animals. The resulting “families” were analyzed separately and genotypes were imputed with MERLIN [39]. Breaking the pedigree in this way produces some loss of information, but simulation results (data not shown) suggested that the loss was negligible.

For experimental data, F_2_ genotypes of non-typed markers were imputed using the algorithm built into the software AlphaImpute [4]. The algorithm implemented in AlphaImpute [4] uses information on population-wide and within family LD and it required certain tuning. In particular, we set the core length parameter to 100, 150, 400 and 600 SNP and the tail parameter haplotype to 300, 400, 600 and 800 SNP, respectively. Likewise, genotype error percentage parameter was set to 0%, so as to obtain a high percentage of alleles under the correct phase [46]. The algorithm was run for the entire pedigree as there was no computing restriction in this case.

### Calculation of the accuracy of imputation

Irrespective of data generation (simulation or experimental), the accuracy of genotype imputation in F_2_ individuals for all methods was evaluated using two different statistics. First, the mean of the difference between observed and imputed allelic dosage was calculated [9,13] as follows:

$$\mathrm{IA}=1-\frac{1}{2N}\sum_{i}^{N}\sum_{j}^{M_{i}}\left|{\hat{g}}_{\mathrm{ij}}-{\hat{g}}_{\mathrm{ij}}\right|$$

In this expression, *N* is the total number of animals imputed, *M*_*i*_ represents the number of markers with observed genotype in animal *i*, *g*_ij_ is the observed (experimental or simulated) allelic dosage in animal *i* and SNP *j,* and ${\hat{g}}_{\mathrm{ij}}$ is the corresponding imputed allelic dosage. Allelic dosage was defined as the number of copies of a reference allele that took values 0, 1 and 2 for homozygous reference, heterozygous and homozygous non-reference, respectively. The second expression used to quantify the imputation accuracy was the square of the correlation between observed and imputed genotypes at each allele, or *R*^2^ statistics of Huang et al. [47]. Denoting $\bar{\hat{g}}$, the average value of the imputed genotypes, and with $\bar{g}$ the average value of observed genotypes, the *R*^2^ statistics were calculated as follows:

R2=∑i=1Ng^ij−g^¯g−ijg¯∑i=1Ng^ij−g^¯2∑i=1Ngij−g¯22

The statistic is interpreted as a squared correlation coefficient.

## Authors’ contributions

JPS, RJCC, JLGD: performed and supervised statistical and simulation analyses and wrote the manuscript. ROB, CWE: designed the resource population and led collection of phenotypic data. CWE, NER: performed DNA extraction and coordinated genotyping with commercial laboratory. JPS, ROB, CWE: designed high density genotyping scheme. All authors read and approved the paper.
